# An unusual case of finger swelling: A case report

**DOI:** 10.1186/1757-1626-2-155

**Published:** 2009-10-16

**Authors:** Richard M Graham, Melanie C Sharp, G Patrick Ashcroft

**Affiliations:** 1 Department of Oral and Maxillofacial Surgery, The Royal Preston Hospital, Sharoe Green Road, Fulwood, Preston, Lancashire, PR2 9HT, UK; 2 Department of Neurosurgery, Nottingham University Hospitals NHS Trust, Queen's Medical Centre campus, Derby Road, Nottingham, NG7 2UH, UK; 3 Department of Orthopaedics, Aberdeen Royal Infirmary, Foresterhill, Aberdeen, AB25 2ZN, UK

## Abstract

A 66 year old man initially presented with haemoptysis and subsequently required a pneumonectomy for a lung mass, following this he had a finger swelling which was found to be a rare leiomyosarcoma and this was a metastatic deposit. This pattern of metastasis for this type of tumour has not been described before.

## Case presentation

**Figure 1:**
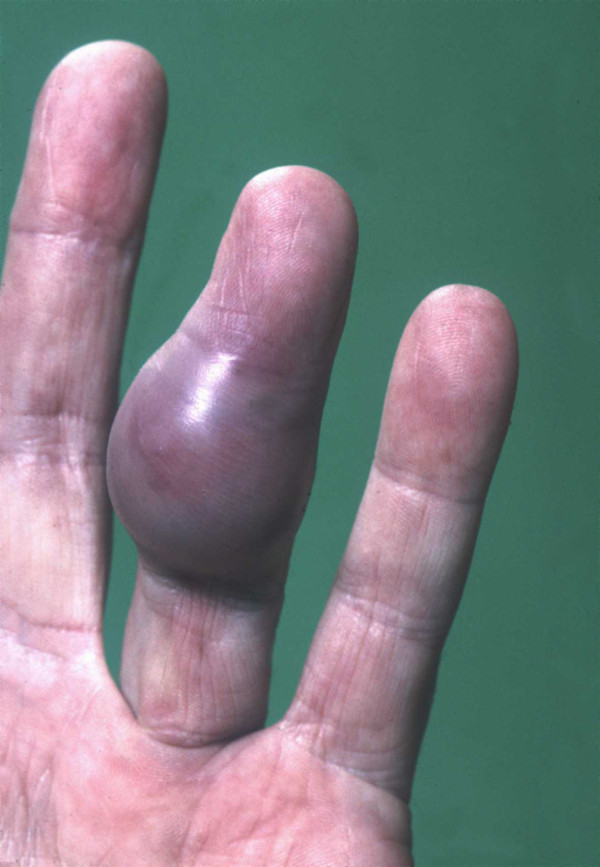
**A clinical image of the finger swelling**. Note the smooth contour, pigmented appearance and a scar on the surface of the swelling, towards the medial aspect.

A 66-year-old man, with a previous left ring finger ganglion, presented with haemoptysis. Radiographic investigations revealed a left lung mass; he underwent a left pneumonectomy for this. The lesion was found to be a rare pulmonary leiomyosarcoma. No further treatment was required, until he was referred to Orthopaedic Surgery, with a possible recurrent left ring finger ganglion (see Figure 1.). This was excised and also found to be a leiomyosarcoma; he therefore had a ray amputation. The sequence of events suggested a primary pulmonary leiomyosarcoma with a metastatic digit deposit, which is extremely rare. Primary leiomyosarcoma of the uterus with pulmonary metastases is a more common presentation [1]. He was referred to Oncology and further limb leiomyosarcomas were found. He therefore had chemotherapy, but died soon afterwards with metastatic leiomyosarcoma. So appearances are not always what they seem.

## Consent

Written informed consent could not be obtained because the patient was deceased. We believe this case report holds a worthwhile clinical lesson which could not be communicated effectively in any other way. Every effort has been made to keep the patient's identity anonymous. We would not expect the patient or their family to object to publication.

## Competing interests

The authors declare that they have no competing interests.

## Authors' contributions

RMG and MCS analysed and interpreted the patient data from the case notes. GPA provided the clinical image and was a major contributor in both suggesting the report and reviewing the manuscript. All authors read and approved the final manuscript.
